# Exploitation of Three Non-Conventional Yeast Species in the Brewing Process

**DOI:** 10.3390/microorganisms7010011

**Published:** 2019-01-08

**Authors:** Laura Canonico, Edoardo Galli, Enrico Ciani, Francesca Comitini, Maurizio Ciani

**Affiliations:** 1 Dipartimento Scienze della Vita e dell’Ambiente, Università Politecnica delle Marche, Via Brecce Bianche, 60131 Ancona, Italy; l.canonico@univpm.it (L.C.); edogah@hotmail.it (E.G.); f.comitini@univpm.it (F.C.); 2 Birra dell’Eremo, microbrewery, Via Monte Peglia, 5, 06081 Assisi, PG, Italy; enrico@birradelleremo.it

**Keywords:** non-conventional yeasts, yeast interactions, beer, bioflavor

## Abstract

Consumers require high-quality beers with specific enhanced flavor profiles and non-conventional yeasts could represent a large source of bioflavoring diversity to obtain new beer styles. In this work, we investigated the use of three different non-conventional yeasts belonging to *Lachancea thermotolerans*, *Wickerhamomyces anomalus*, and *Zygotorulaspora florentina* species in pure and mixed fermentation with the *Saccharomyces cerevisiae* commercial starter US-05. All three non-conventional yeasts were competitive in co-cultures with the *S. cerevisiae*, and they dominated fermentations with 1:20 ratio (*S. cerevisiae*/non-conventional yeasts ratios). Pure non-conventional yeasts and co-cultures affected significantly the beer aroma. A general reduction in acetaldehyde content in all mixed fermentations was found. *L. thermotolerans* and *Z. florentina* in mixed and *W. anomalus* in pure cultures increased higher alcohols. *L. thermotolerans* led to a large reduction in pH value, producing, in pure culture, a large amount of lactic acid (1.83 g/L) while showing an enhancement of ethyl butyrate and ethyl acetate in all pure and mixed fermentations. *W. anomalus* decreased the main aroma compounds in comparison with the *S. cerevisiae* but showed a significant increase in ethyl butyrate and ethyl acetate. Beers produced with *Z. florentina* were characterized by an increase in the isoamyl acetate and α-terpineol content.

## 1. Introduction

In the brewing industry, *Saccharomyces cerevisiae* and *Saccharomyces pastorianus* are the most used yeast species in starter cultures ensuring certain advantages in the fermentation process and in the standard product quality. In the past few years, brewers have payed attention to yeast selection not only for their fermentation efficiency and technological advantages but also for the aromatic characters and the flavors they give to the final product. To this end, recent genetic investigations have focused on methods to enhance the fermentation efficiency and aromatic profile of selected *S. cerevisiae* strains [1,2,3]. Other studies proposed the isolation of new starter yeasts (*S. cerevisiae*) from natural matrices [4,5] and the selection of wine yeast strains [6]. In recent years, the rapid expansion in the number of microbreweries has led the brewers to differentiate their products through the use of alternative raw materials and the selection and use of non-conventional yeasts [7,8,9,10]. Indeed, bioflavor from metabolic pathways of yeasts may be a suitable strategy to obtain beer with a different aromatic taste [11,12]. In this context, the use of non-conventional yeasts could be an alternative way to enhance the aroma profile of beers. Within non-conventional yeasts, different genera and species have been proposed. *Torulaspora delbrueckii* is a yeast species widely studied in winemaking for its ability to produce fruitiness and positive aromatic flavor, and for this reason, it was also evaluated for beer production, both in pure and in mixed cultures with different *S. cerevisiae* starter strains [7,13]. Beers produced with pure cultures of *T. delbrueckii* were characterized by a low alcohol content (2.66% *v*/*v*) but at the same time with distinctive analytical and aromatic profile [7,14]. *Lachancea termotholerans*, another non-conventional yeast species, was proposed by Domizio et al. [15] to produce sour beers without the use of lactic acid bacteria. Indeed, they investigated the use of a strain of *L. thermotolerans* in pure culture able to decrease the pH value and increase the glycerol and lactic acid production. Other non-conventional yeasts belonging to the *Hanseniaspora vineae*, *Lachancea fermentati*, *Schizosaccharomyces japonicus*, and *Wickerhamomyces anomalus* species showed promising fermentation aptitude and sensory features for the production of sour beer [8]. In addition, yeasts belonging to *Cyberlindnera fabianii*, *Pichia kudriavzevii,* and *Pichia kluyverii* were evaluated to tailor the aroma production and the ethanol content in beer [16,17].

In the present study, after a preliminary screening of yeasts from the Collection of the Department of Life and Environmental Sciences (DiSVA), we evaluated the use of selected strains of *L. thermotolerans*, *W. anomalus*, and *Zygotorulaspora florentina* in pure and in mixed fermentation at different inoculation ratios with *S. cerevisiae* in the brewing process. The yeast strains’ population dynamic and their influence on the bioflavor of beer were evaluated.

## 2. Materials and Methods

### 2.1. Yeast Strains

The yeast strains used in this study were six strains of *L. thermotolerans*, nine strains of *W. anomalus* and eight strains of *Z. florentina* coming from the Yeast Collection of (DiSVA) of the Polytechnic University of Marche (Italy). The *S. cerevisiae* commercial strain US-05 (Fermentis, Lesaffre, France) was used in the mixed fermentation trials and as the control. The US-05 was rehydrated following the manufacturer’s instructions.

### 2.2. Preliminary Screening and Fermentation Trials

The fermentation of maltose by these non-conventional yeasts strains was assessed in flasks containing 100 mL of malt extract 10%. The capacity to utilise the maltose was determined by measuring the weight loss of the flasks due to the CO_2_ evolution, which was followed to the end of the fermentation (i.e., constant weighing for 3 consecutive days). From this preliminary screening, the strains able to ferment maltose were selected and used in pure and mixed fermentations with the *S. cerevisiae* US-05 starter strain at different ratios of *S. cerevisiae* to non-*Saccharomyces* (i.e., 1:1, 1:10, 1:20). The fermentation performances of the strains in pure and mixed fermentations were evaluated in 500-mL flasks containing 500 mL of wort at 19 ± 1 °C and inoculated by pre-cultures grown in 10% malt extract at 19 ± 1 °C for 48 h, locked with a Müller valve containing sulphuric acid, to allow the CO_2_ to escape from the system in the same conditions reported by Canonico et al. [7,13].

The populations dynamic was monitored during the fermentation process collecting the samples at established intervals. One hundred µL aliquots of serial dilutions of each sample were plated onto both WL nutrient Agar (Oxoid, Hampshire, UK) and Lysine Agar (Oxoid, Hampshire, UK) able to differentiate non-*Saccharomyces* yeast population from the US-05 *S. cerevisiae* starter strain as reported by Canonico et al. [7,13]. The fermentations were carried out in triplicate trials under static conditions.

### 2.3. Wort Production

The wort used for the micro fermentation trials was made with 100% pilsner malt and the Cascade hop variety by Birra dell’Eremo Microbrewery (Assisi, Italy). The wort was produced from a batch of 1500 L following these mashing steps: 53 °C for 10 min, 67 °C for 70 min, and 76 °C for 10 min, with boiling for 60 min. The wort obtained showed the following main analytical characters: pH 5.47; specific gravity 12.3° Plato (original gravity of 1.050); free assimilable nitrogen, 263 mg N/L, and 20 IBU (International Bitterness Unit).

### 2.4. Analytical Procedures

The Official European Union Methods [18] were used to determine the ethanol content, volatile acidity, and pH. Acetaldehyde, ethyl acetate, n-propanol, isobutanol, amyl, and isoamyl alcohols, and acetoin were quantified by direct injection into a gas-liquid chromatography system (GC-2014; Shimadzu, Kjoto, Japan). Each sample was prepared and analysed as reported by Canonico et al. [19].

The solid-phase microextraction (HS-SPME) method was used to determine the concentration of the volatile compounds. Five mL of beer was placed in a vial containing 1 g NaCl closed with a septum-type cap. HS-SPME was carried out with magnetic stirring for 10 min at 25 °C. After this period, the internal standard (3-octanol) at a concentration of 1.6 mg/L was added, and the sample was heated to 40 °C. Divinylbenzene/carboxen/polydimethylsiloxane (DVB/CAR/PDMS) fibre (Sigma-Aldrich) was inserted into the vial headspace for 30 min. The compounds were desorbed by inserting the fibre into a Shimadzu gas chromatograph GC injector for 5 min. A glass capillary column was used: 0.25 μm Supelcowax 10 (length, 60 m; internal diameter, 0.32 mm). The fibre was inserted in the split–splitless mode as reported by Canonico et al. [19]. The compounds were identified and quantified by comparisons with external calibration curves for each compound.

Final gravity was determined by a densimeter (Polsinelli Enologia Srl, Isola Liri, Italy) at a temperature of 20 °C. Specific enzymatic kit (Megazyme, Wicklow, Ireland) was used to determine the concentration of lactic acid (kit K-DLATE) according to the manufacturer instructions.

### 2.5. Statistical Analysis

Analysis of variance (ANOVA) was applied to the experimental data for the main characteristics of the beers. The means were analysed using STATISTICA 7 software. Significant differences were determined by the means of Duncan tests, and the results were considered significant if the associated *p* values were <0.05. Principal component analysis (PCA) was applied to discriminate between the means of the contents of esters, higher alcohols, and carbonyl compounds in the beers from the pure and mixed fermentations PCA, which was carried out using the statistical software package JMP 11^®^. The mean data were normalised to neutralize any influence from hidden factors. The PCA provides a graphical representation of the overall differences due to the non-*Saccharomyces* in terms of fermenting products of the final beers.

## 3. Results

### 3.1. Preliminary Screening

Initial screening of the strains belonging to the different species was carried out to determine their ability to ferment maltose, the most abundant fermentable sugar in the brewing wort, to select for their potential use in beer production (Table 1). The results of the fermentation capacity showed that all of the strains tested exhibited the ability to ferment maltose, although even at different grades. From the strains tested, we chose *L. thermotolerans* DISVA 322, *Z. florentina,* DiSVA 263, and *W. anomalus* DiSVA 2 since they showed the best fermentative performance with the highest final CO_2_ evolved. Regarding the species *W. anomalus*, the data reported in Table 1 highlighted that different strains belonging to this species exhibited a good fermentation performance. The choice to select *W. anomalus* DiSVA 2 has been evaluated taking into account the screening and the results reported by Oro et.al [20] regarding the metabolic activities and aromatic compounds production. For these reasons, these three strains were selected for the subsequent micro-fermentation trials in pure and in co-culture at different inoculation ratios *S. cerevisiae*/non-conventional yeasts (1:1; 1:10; 1:20) using the *S. cerevisiae* starter strain US-05.

### 3.2. Fermentation Trials with the Selected Non-Saccharomyces Yeasts

#### 3.2.1. Evaluation of Population Dynamics

**Figure 1:**
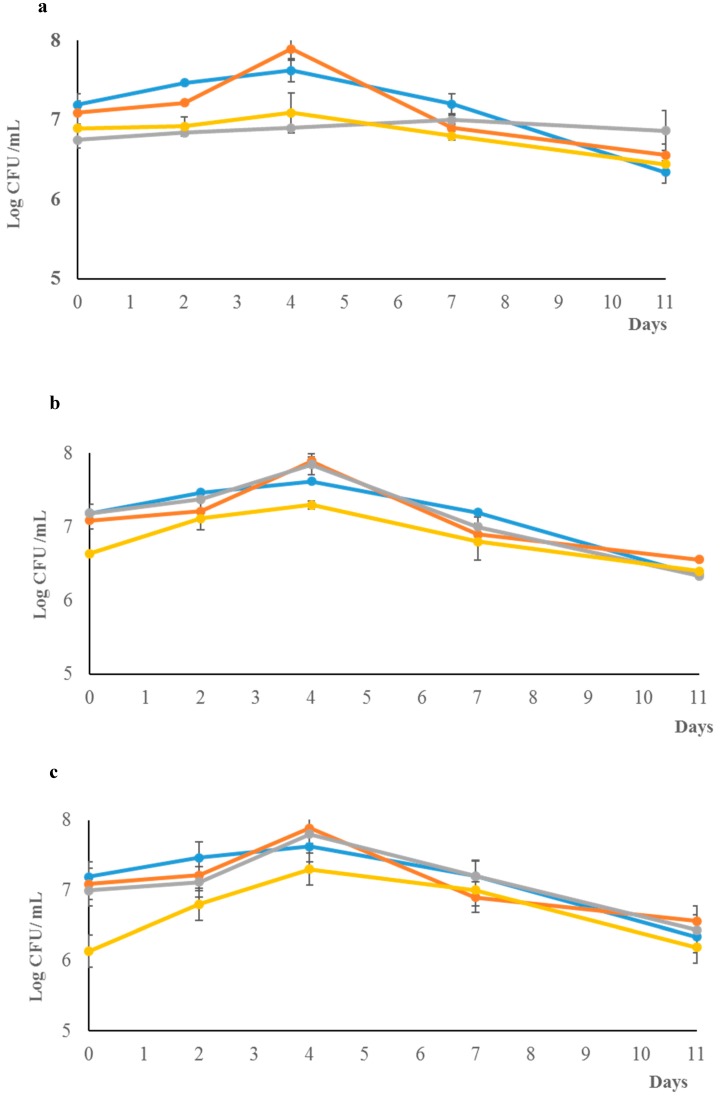
Growth kinetics of *L. thermotolerans* in pure and mixed fermentation. Pure cultures of *L. thermotolerans* (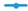) and *S. cerevisiae* (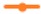) and for the mixed fermentations with *S. cerevisiae* (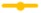) and *L. thermotolerans* (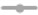) individually in the mixed cultures at 1:1 (**a**), 1:10 (**b**) and 1:20 (**c**).

The growth kinetics of *L. thermotolerans* in pure and in mixed fermentation are reported in Figure 1. The *S. cerevisiae* US-05 pure cultures achieved 10^8^ CFU/mL after four days of fermentation (maximum viable cells) and maintained 10^7^ CFU/mL until the end of the fermentation. Differently, in the mixed cultures with the inoculation ratio of 1:1 (Figure 1a), *S. cerevisiae* US-05 reached a lower biomass, achieving only 10^7^ CFU/mL on the fourth day of fermentation. The same trend was also exhibited for *S. cerevisiae* US-05 with the inoculation ratios of 1:10 and 1:20 (Figure 1b,c). *S. cerevisiae* was not inhibited at all in the inoculation ratios and completed fermentation as showed by residual sugar and ethanol content in the final beers (Table 2).

*L. thermotolerans* in mixed fermentation at the 1:10 and 1:20 inoculation ratios showed a biomass evolution comparable with that of the pure culture, while at the 1:1 inoculation ratio, *L. thermotolerans* showed a very limited increase in biomass compared with that exhibited by *S. cerevisiae* under the same conditions (Figure 1a). Under these conditions, both strains suffered a mutual inhibition but showed no differences compared with the control trial (US 05 strain) (Table 2).

**Figure 2:**
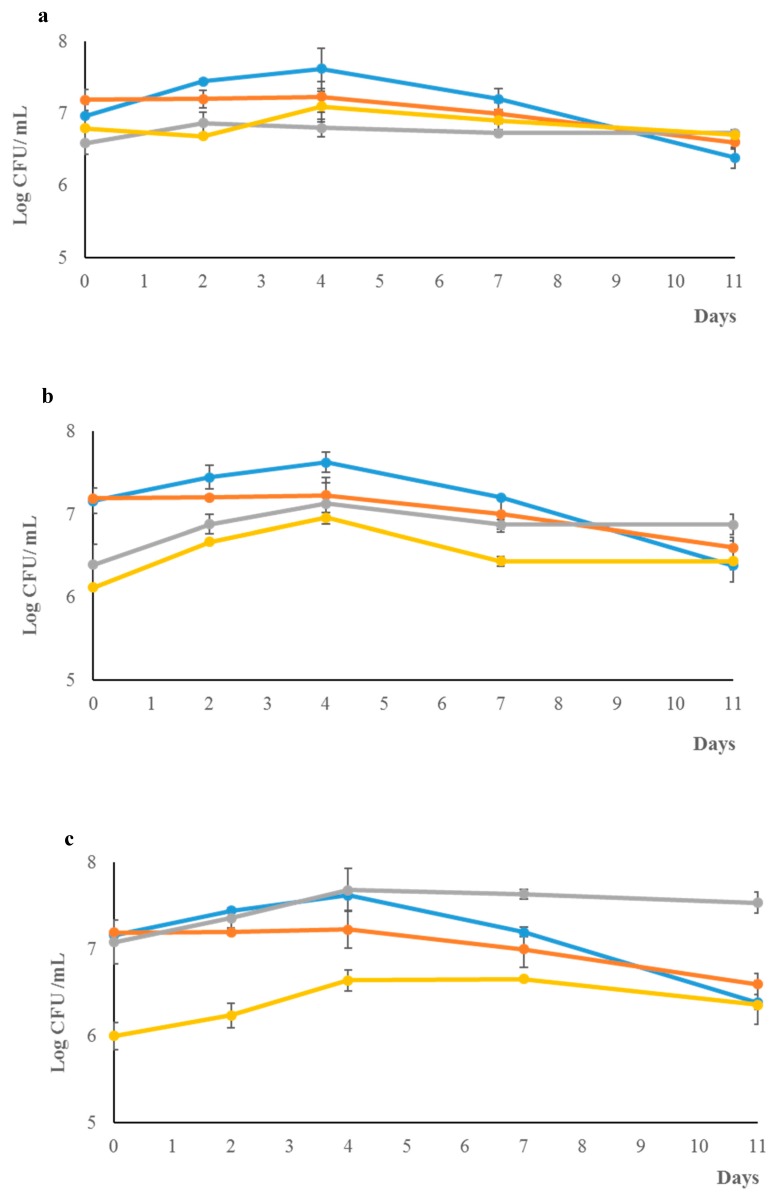
Growth kinetics of *Z. florentina* in pure and mixed fermentation. Pure cultures of *Z. florentina* (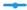) and *S. cerevisiae* (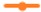) and for the mixed fermentation with. *S. cerevisiae* (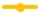) and *Z. florentina* (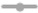) individually in the mixed cultures at 1:1 (**a**), 1:10 (**b**) and 1:20 (**c**).

The biomass evolution of the *S. cerevisiae* and *Z. florentina* mixed fermentations at the 1:1 inoculation ratio (Figure 2a) exhibited a similar growth kinetic (with a slight prevalence of *S. cerevisiae*), and they reached a maximum cell concentration of 10^7^ mL/L after four days, showing a reciprocal inhibition compared with pure cultures.

At the 1:10 and 1:20 inoculation ratios (Figure 2a,b), the two yeast species did not show the antagonistic effect on each other, however, *Z. florentina* prevailed over *S. cerevisiae* during the fermentation processes.

**Figure 3:**
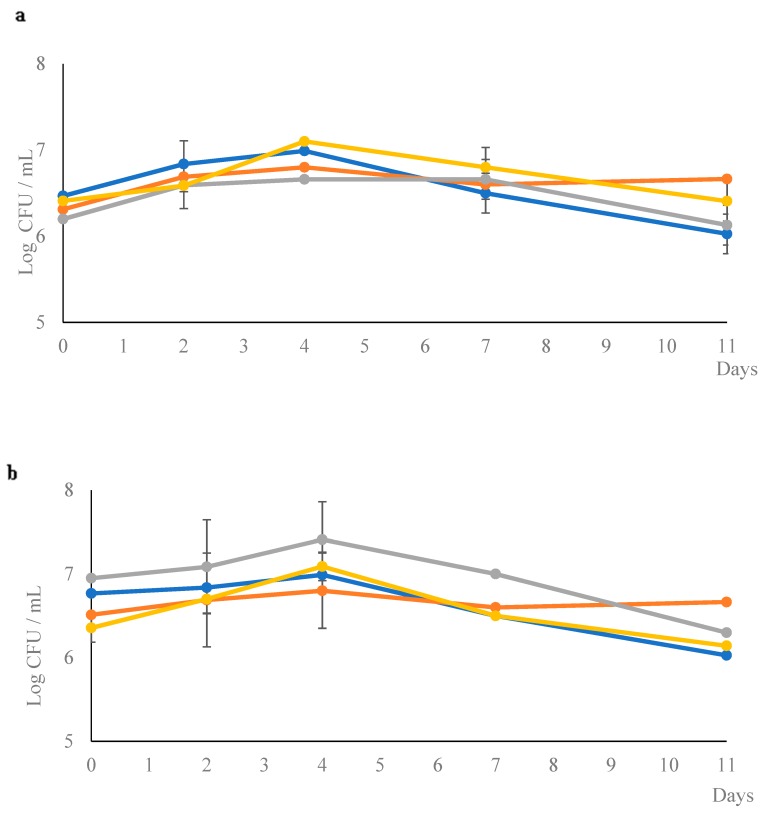

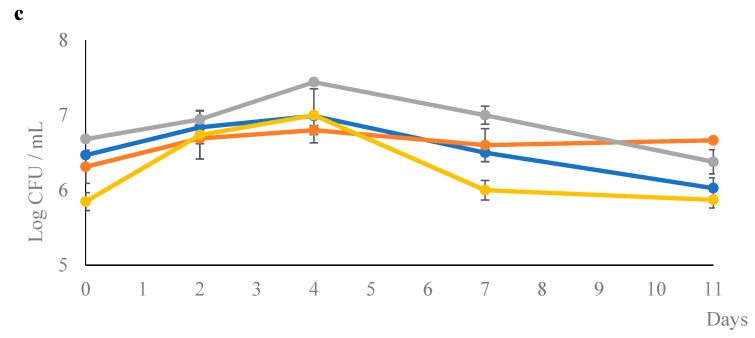
Growth kinetics of *W. anomalus* in pure and mixed fermentation. Pure cultures of *W. anomalus* (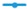) and *S. cerevisiae* (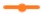) and for the mixed fermentation with *S. cerevisiae* (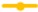) and *W. anomalus* (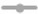) individually in the mixed cultures at 1:1 (**a**), 1:10 (**b**) and 1:20 (**c**).

The cell evolution of *W. anomalus* in pure and mixed fermentations with *S. cerevisiae* showed the same trend exhibited by *Z. florentina* (Figure 3), indicating a good competition with *S. cerevisiae* in wort fermentation, especially when the inoculation level was 10- or 20-fold higher (1:10 and 1:20 trials). Despite the competition shown by the three non-conventional yeast species toward *S. cerevisiae*, all mixed fermentation trials (at different inoculation ratios) did not show relevant differences in the main analytical characteristics in comparison with the *S. cerevisiae* control trial (Table 2). Therefore, all the mixed fermentations have completed the fermentation as attested by the values of final gravity that are comparable with *S. cerevisiae* pure culture (Table 2).

#### 3.2.2. Main Analytical Profiles

The data regarding the analytical compositions of the beers produced are reported in Table 2.

The pure cultures with non-conventional yeasts showed significant reductions in ethanol content compared with the *S. cerevisiae* starter strain (4.03% *v*/*v*). Indeed, beers produced by pure cultures of *L. thermotolerans, Z. florentina*, and *W. anomalus* exhibited an ethanol content of 3.12% *v*/*v*, 3.47% *v*/*v*, and 1.53% *v*/*v*, respectively. Consequently, pure cultures of the non-conventional yeasts showed higher final gravities. On the other hand, all mixed cultures produced beers with an ethanol content not significantly different to that of the *S. cerevisiae* US-05 control.

For the volatile acidity, the results did not show significant differences for the trials carried out with *L. thermotolerans* in comparison with *S. cerevisiae* pure culture, while *Z. florentina* at inoculation ratio 1:20 exhibited a significant increase in acetic acid content in comparison with *S. cerevisiae* and the other trials. Regarding *W. anomalus* significant increase was exhibited in pure and at inoculation ratio 1:20.

All of the beers exhibited pH values comparable to that of the *S. cerevisiae* starter strain, with the only exception being *L. thermotolerans* in pure and mixed fermentations. In particular, *L. thermotolerans* in pure culture exhibited a pH reduction of ca. 0.6 points. Regarding the lactic acid content, *L. thermotolerans* in pure and at different inoculum ratio exhibited a significantly higher concentration in comparison with the *S. cerevisiae* and the other two non-conventional yeasts. On the other hand, *W. anomalus* and *Z. florentina* in pure cultures exhibited lactic acid amounts significantly higher than those shown by the *S. cerevisiae*. Moreover, *Z. florentina* also at inoculation ratio 1:1 and 1:20 (*S. cerevisiae*/*Z. florentina*) exhibited a significant increase in comparison with *S. cerevisiae*, while at 1:10 ratio showed a comparable amount of lactic acid of *S. cerevisiae*. All mixed fermentation carried out with *W. anomalus* significantly increased the lactic acid content in comparison with *S. cerevisiae*.

#### 3.2.3. Main Volatile Compounds

The main volatile compounds produced by *L. thermotolerans* in pure and mixed fermentations are reported in Table 3. The results highlighted a significant increase in ethyl acetate (fruit notes) content in pure and mixed fermentations compared with *S. cerevisiae. L. thermotolerans* in pure and mixed fermentations showed a significant increase in ethyl butyrate content, which is the ester responsible for the fruity or solvent aroma of beer [21], compared with *S. cerevisiae* US-05. A significant increase in isoamyl acetate content (banana aroma) was exhibited by *L. thermotolerans* at the 1:20 *S. cerevisiae: L. thermotolerans* inoculation ratio compared with *S. cerevisiae* in pure culture, while the concentration of ethyl hexanoate (i.e., apple, fruit flavour) decreased with increasing the inoculation ratios. The same trend was also exhibited for the linalool content. Furthermore, the citronellol content decreased in mixed fermentation at the 1:10 and 1:20 inoculation ratios, while at the 1:1 its concentration was comparable with that exhibited by the *S. cerevisiae* starter strain. Moreover, the mixed cultures showed significant increases in higher alcohols levels with the exception of β-phenyl ethanol. In regard to the acetaldehyde content, the mixed fermentations at the 1:1 inoculation ratio exhibited a reduction compared with the other beers.

In mixed fermentation trials, *Z. florentina* showed a significant decrease in acetaldehyde content compared with the beer obtained with the *S. cerevisiae* starter strain (Table 4).

Regarding the ester compounds, Table 4 showed a significant increase in ethyl butyrate content at 1:20 inoculation ratio in comparison with the other trials, while ethyl acetate increases in all mixed fermentation, phenyl ethyl acetate, ethyl hexanoate, and isoamyl acetate decreases when raising the inoculation ratio. Moreover, the mixed fermentations showed a significant increase in some higher alcohols content, with the only exception of β-phenyl ethanol that decreased in mixed fermentation in comparison with *S. cerevisiae* pure culture. Regarding terpens compounds, no significant differences were exhibited for α-terpineol, while citronellol content significant decrease in mixed fermentation in comparison with *S. cerevisiae* pure culture.

The use of *W. anomalus* (Table 5) in mixed fermentation led to a reduction in acetaldehyde and an increase in ethyl acetate in comparison with the beers obtained with *S. cerevisiae*. In pure fermentation, *W. anomalus* showed a significant increase of higher alcohols (n-propanol, isobutanol, amylic and isoamylic alcohol). On the other hand, *W. anomalus* in mixed fermentations showed higher alcohols concentrations comparable to those exhibited by *S. cerevisiae*. *W. anomalus* in mixed fermentations increased the ethyl butyrate content, while ethyl hexanoate, phenyl ethyl acetate, and β-phenyl ethanol were decreased in comparison with *S. cerevisiae* pure culture. In contrast, the levels of linalool did not show significant differences in the final beers. Regarding α-terpineol, this aroma compound significantly increased in mixed fermentation at the 1:1 inoculation ratio. The citronellol content decreased in mixed fermentations with increasing of the inoculation ratio of *W. anomalus*.

**Figure 4:**
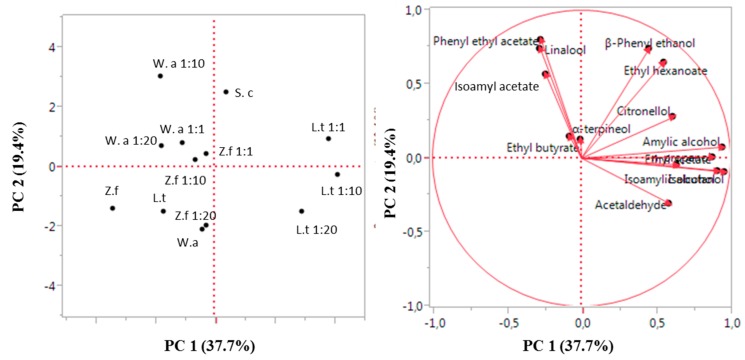
Principal component analysis for the main by-products and volatile compounds of the pure non-conventional yeasts (L. t = *L. thermotolerans*; W. a = *W. anomalus*; Z. f = *Z. florentina*) and their mixed fermentations with *S. cerevisiae* (S. c). The variance explained by principle component analysis (PCA) analysis is PC 1 37.7% X-axis and PC 2 19.4% Y-axis). PC 1: principal component.

To assess the overall effects of non-conventional yeasts in pure and mixed fermentation, data regarding all of the volatile compounds were analysed by PCA (Figure 4). The PCA analysis showed that *Z. florentina* in pure and mixed fermentation fell into the left half of sample distribution together with *L. thermotolerans* and *W. anomalus* pure cultures. Moreover, *W. anomalus* and *L. thermotolerans* mixed fermentations are located in the upper right, and in the right of the representation respectively. These results highlighted that each non-conventional yeast species affect the final volatile profile in a different way.

## 4. Discussion

In winemaking, the controlled use of non-conventional yeast species has been investigated to enhance complexity and give distinctive flavour profiles of wines [22,23,24].

Indeed, fermented alcoholic beverages with peculiar and distinctive flavours can be obtained through the yeast inoculation during the fermentation process [11,21,25]. In this regard, the potential use of non-conventional yeasts in brewing has been poorly explored. Only recently, some studies focused on flavouring by non-conventional yeasts in brewing [7,9,14,16,26].

In this work, we investigated three non-conventional yeast species widely found in the wine environment [23,27,28]. The three strains chosen within the three species were competitive versus the *S. cerevisiae* starter strain at the 1:1 inoculation ratio exhibiting similar numbers of viable cells. The competition of the non-conventional species in wort under semi-anaerobic conditions was confirmed for the 1:10 and 1:20 ratios (*S. cerevisiae* brewers’ yeast: Non-conventional species). In these last conditions, all three non-conventional yeast species coexisted with brewers’ yeast dominating the fermentation process. Similar results were found by Van Rijswijck et al. [17] where *Candida fabianii* at a 1:100 ratios (*S. cerevisiae*: *C. fabianii*) dominated the fermentation. However, compared to the results of the present work, a large amount of residual maltose was found.

This behaviour is of interest from a practical application point of view. Indeed, the dominance allows displaying the metabolism of the non-conventional yeast in co-culture with the brewing starter, obtaining, at the same time, a distinctive aromatic impact on final product and a correct and complete evolution of the fermentation. In this regard, differences in the analytical composition using the three non-conventional yeasts in pure and in co-culture were found (see Figure 4). All three non-conventional species determined a general decrease of acetaldehyde in all mixed fermentations (1:1, 1:10, 1:20 *S. cerevisiae*/non-conventional yeasts ratios).

*L. thermotolerans*, a yeast species widely investigated in wine fermentation, [28,29] showed a large reduction in pH compared with the *S. cerevisiae* starter strain. Domizio et al. [15] and Osburn et al. [8], investigating *L. thermotolerans* in pure fermentation with the goal of producing sour beer, and found a compared trend in pH reduction. In the wort used in this work, *L. thermotolerans* in pure culture produced a larger amount of lactic acid (1.83 g/L) compared with the quantities shown in the study of Domizio et al. [15] (0.24 g/L). Regarding the compounds influencing the bioflavor, *L. thermotolerans* produced beer with a higher ethyl butyrate content and an increase in ethyl acetate (i.e., floral, honey, sweet) in pure and in all of the mixed fermentations compared with *S. cerevisiae*. Moreover, a significant increase in isoamyl acetate content (banana aroma) was exhibited in the trial *S. cerevisiae*/*L. thermotolerans* at an inoculum ratio of 1:20. These results highlighted that this strain was interesting not only for sour beer production due to its lactic acid production and consequent pH decrease during primary fermentation but also to tailor the aroma profile of the beer. The low ethanol content exhibited by *L. thermotolerans* pure cultures was also relevant for the production of low alcohol beer. *W. anomalus* generally studied in apple wine and hard cider production [30,31], it was poorly investigated in brewing because it is associated with the concept of “spoilage yeast” [32]. Only Osburn at al. [8] showed that a strain of *W. anomalus* exhibited a good fermentation performance and led beers with fruit notes such as apple, pear, and apricot. Results of this study showed an increase in ethyl butyrate and ethyl acetate and a reduction in acetaldehyde in all mixed fermentations that positively affect the aromatic profile of the beer. As reported above, there are no studies on the use of this species in beer, thus, for this reason, it is difficult to understand the general behaviour of this non-conventional yeast in the brewing process and further investigations are needed. Furthermore, there is only one study that evaluated the use of the non-conventional yeast *Z. florentina* in brewing. In contrast to the report of Holt et al. [16] that showed increases in higher alcohols, we observed a different trend for these compounds. On the other hand, *Z. florentina* showed increased isoamyl acetate and α-terpineol content (floreal aroma) that positively influenced the final flavour of the beer. In conclusion, the use of non-conventional yeasts in mixed fermentations with *S. cerevisiae* is a suitable strategy to tailor flavour production during beer fermentation, thus making it possible to obtain products with aroma compounds that are different from those of beers brewed using pure *S. cerevisiae* starter strains. These data confirm that the brewing yeast used can modulate the production of the aroma compounds in the final beers. The modalities (pure or co-cultures with *S. cerevisiae*) and the inoculation ratio in mixed fermentations should be further investigated, even if the non-conventional yeasts coexist, and in some cases, dominate the process, the fermentations are almost completed. In this regard, further work is needed to understand the behaviours of these non-conventional yeasts in the brewing process and to know their possible uses in different beer styles. The next necessary step will be evaluating the selected combination of *S. cerevisiae*/non-conventional yeasts on a large scale to evaluate through sensory analysis the beer style more suitable for these yeasts.

## Figures and Tables

**Table 1:** Fermentation parameters of different non-conventional yeasts on malt extract.

Strains	Total CO_2_ g Evolved (20 Days)	Fermentation Rate (g CO_2_/Day)
*L. thermotolerans* DiSVA 322	3.87 ± 0.05	0.31 ± 0.05
*L. thermotolerans* DISVA 324	3.49 ± 0.04	0.16 ± 0.03
*L. thermotolerans* DiSVA 325	2.04 ± 0.37	0.13 ± 0.06
*L. thermotolerans* DiSVA 326	2.13 ± 0.20	0.10 ± 0.05
*L. thermotolerans* DiSVA 327	3.60 ± 0.08	0.16 ± 0.05
*W. anomalus* DiSVA 2	1.68 ± 0.40	0.22 ± 0.09
*W. anomalus* DiSVA 45	1.30 ± 0.21	0.17 ± 0.06
*W. anomalus* DiSVA 45	1.84 ± 0.12	0.23 ± 0.07
*W. anomalus* DiSVA 56	1.58 ± 0.06	0.16 ± 0.03
*W. anomalus* DiSVA 336	1.27 ± 0.03	0.18 ± 0.03
*W. anomalus* DiSVA 355	1.47 ± 0.12	0.19 ± 0.04
*W. anomalus* DiSVA 356	1.58 ± 0.35	0.21 ± 0.10
*W. anomalus* DiSVA 359	1.40 ± 0.25	0.18 ± 0.02
*W. anomalus* DiSVA 383	1.69 ± 0.07	0.22 ± 0.05
*W. anomalus* DiSVA 384	1.45 ± 0.09	0.20 ± 0.06
*W. anomalus* DiSVA 385	1.71 ± 0.14	0.20 ± 0.06
*W. anomalus* DiSVA 386	1.82 ± 0.11	0.23 ± 0.06
*Z. florentina* DiSVA 262	3.41 ± 0.12	0.34 ± 0.02
*Z. florentina* DiSVA 263	3.66 ± 0.13	0.32 ± 0.06
*Z. florentina* DiSVA 264	2.33 ± 0.14	0.14 ± 0.03
*Z. florentina* DiSVA 309	2.52 ± 0.21	0.23 ± 0.05
*Z. florentina* DiSVA 310	1.14 ± 0.36	0.16 ± 0.05
*Z. florentina* DiSVA 311	1.16 ± 0.09	0.23 ± 0.09
*Z. florentina* DiSVA 312	1.84 ± 0.14	0.27 ± 0.07
*Z. florentina* DiSVA 317	0.68 ± 0.31	0.01 ± 0.01
*S. cerevisae* US-05	4.61 ± 0.05	0.65 ± 0.04

Data are the means ± standard deviations. CO_2_ g evolved after 20 days of fermentation (in 100 mL of 10% malt extract). Fermentation rate: CO_2_ g/day (over the first 6 days of fermentation).

**Table 2:** The main analytical characteristics of the beer produced by the pure and mixed fermentations.

Fermentation	Ethanol (% *v*/*v*)	Residual Sugar g/L (Maltose)	Final Gravity	Apparent Attenuation (%)	Real Attenuation (%)	Volatile Acidity (g/L)	pH	Lactic Acid (g/L)
***L. thermotolerans*** **DiSVA 322**	3.12 ± 0.32 ^b^	12.32 ± 0.03 ^a^	1.023 ± 0.17 ^b^	48.95 ± 1.47 ^c^	39.80 ± 1.20 ^c^	0.38 ± 0.00 ^a^	3.88 ± 0.03 ^b^	1.83 ± 0.07 ^a^
**0.0US05 + DiSVA 322 1:1**	4.03 ± 0.01 ^a^	0.07 ± 0.03 ^b^	1.015 ± 0.0 ^c^	68.75 ± 0.00 ^b^	55.90 ± 0.00 ^b^	0.34 ± 0.00 ^a^	4.23 ± 0.18 ^a^	0.53 ± 0.05 ^c^
**US05 + DiSVA 322 1:10**	4.09 ± 0.02 ^a^	0.07 ± 0.03 ^b^	1.015 ± 0.00 ^c^	68.75 ± 0.01 ^b^	55.90 ± 0.00 ^b^	0.34 ± 0.00 ^a^	4.19 ± 0.09 ^a^	0.82 ± 0.01 ^b^
**US05 + DiSVA 322 1:20**	4.09 ± 0.23 ^a^	0.02 ± 0.04 ^b^	1.015 ± 0.00 ^c^	68.75 ± 0.02 ^b^	55.90 ± 0.00 ^b^	0.36 ± 0.16 ^a^	4.14 ± 0.00 ^a^	0.85 ± 0.04 ^b^
***S. cerevisiae*** **US05**	4.03 ± 0.11 ^a^	0.05 ± 0.01 ^b^	1.010 ± 0.00 ^a^	79.16 ± 0.00 ^a^	64.36 ± 0.00 ^a^	0.36 ± 0.00 ^a^	4.46 ± 0.12 ^a^	0.01 ± 0.00 ^d^
***Z. florentina*** **DiSVA 263**	3.47 ± 0.15 ^b^	17.32 ± 0.10 ^a^	1.025 ± 0.00 ^b^	47.91 ± 2.94 ^d^	38.95 ± 2.40 ^d^	0.34 ± 0.00 ^b^	4.48 ± 0.02 ^a^	0.21 ± 0.05 ^a^
**US05 + DiSVA 263 1:1**	4.04 ± 0.35 ^a^	0.70 ± 0.01 ^b^	1.020 ± 0.17 ^c^	59.37 ± 1.47 ^c^	48.27 ± 1.20 ^c^	0.48 ± 0.16 ^b^	4.45 ± 0.19 ^a^	0.18 ± 0.00 ^a^
**US05 + DiSVA 263 1:10**	3.81 ± 0.07 ^b^	0.32 ± 0.01 ^b^	1.020 ± 0.00 ^c^	58.33 ± 0.00 ^c^	47.42 ± 0.00 ^c^	0.38 ± 0.00 ^b^	4.53 ± 0.26 ^a^	0.05 ± 0.02 ^b^
**US05 + DiSVA 263 1:20**	4.30 ± 0.07 ^a^	0.02 ± 0.01 ^b^	1.015 ± 0.17 ^d^	68.75 ± 2.94 ^b^	55.89 ± 2.40 ^b^	0.60 ± 0.00 ^a^	4.42 ± 0.16 ^a^	0.16 ± 0.01 ^a^
***S. cerevisiae*** **US05**	4.03 ± 0.11 ^a^	0.05 ± 0.01 ^b^	1.010 ± 0.00 ^a^	79.16 ± 0.00 ^a^	64.36 ± 0.00 ^a^	0.36 ± 0.00 ^b^	4.46 ± 0.12 ^a^	0.01 ± 0.00 ^b^
***W. anomalus*** **DiSVA 2**	1.53 ± 0.10 ^b^	57.32 ± 0.05 ^a^	1.035 ± 0.00 ^a^	27.08 ± 2.94 ^c^	22.02 ± 2.39 ^c^	0.46 ± 0.00 ^a^	4.75 ± 0.02 ^a^	0.17 ± 0.01 ^a^
**US05 + DiSVA 2 1:1**	4.06 ± 0.01 ^a^	0.07 ± 0.01 ^b^	1.015 ± 0.00 ^b^	69.79 ± 0.00 ^b^	55.89 ± 0.00 ^b^	0.22 ± 0.00 ^c^	4.48 ± 0.11 ^a^	0.12 ± 0.01 ^b^
**US05 + DiSVA 2 1:10**	4.01 ± 0.04 ^a^	0.02 ± 0.01 ^b^	1.015 ± 0.17 ^b^	55.89 ± 2.94 ^b^	56.74 ± 2.39 ^b^	0.34 ± 0.00 ^b^	4.44 ± 0.21 ^a^	0.19 ± 0.00 ^a^
**US05 + DiSVA 2 1:20**	3.99 ± 0.47 ^a^	0.02 ± 0.01 ^b^	1.015 ± 0.00 ^b^	55.89 ± 0.00 ^b^	55.89 ± 0.00 ^b^	0.48 ± 0.00 ^a^	4.47 ± 0.15 ^a^	0.19 ± 0.00 ^a^
***S. cerevisiae*** **US05**	4.03 ± 0.11 ^a^	0.05 ± 0.01 ^b^	1.010 ± 0.00 ^a^	79.16 ± 0.00 ^a^	64.36 ± 0.00 ^a^	0.36 ± 0.00 ^b^	4.46 ± 0.12 ^a^	0.01 ± 0.00 ^c^

Data are the means ± standard deviation. Data with different superscript letters (^a,b,c,d^) within each column and each non-conventional species compared to *S. cerevisiae* US05 are significantly different (Duncan tests; *p* < 0.05).

**Table 3:** The main by-products and volatile compounds in the beers produced by *L. thermotolerans* in the pure and mixed fermentations (mg/L).

	*L. thermotolerans* DiSVA 322	US05 + DiSVA 322 1:1	US05 + DiSVA 322 1:10	US05 + DiSVA 322 1:20	*S. cerevisiae* US-05
**Esters**					
Ethyl butyrate	0.32 ± 0.01 ^a^	0.20 ± 0.13 ^ab^	0.14 ± 0.03 ^bc^	0.14 ± 0.03 ^bc^	0.04 ± 0.01 ^c^
Ethyl acetate	17.4 ± 4.6 ^bc^	21.3 ± 0.6 ^a^	17.0 ± 4.4 ^bc^	24.6 ± 6.7 ^a^	2.6 ± 0.2 ^c^
Phenyl ethyl acetate	0.01 ± 0.01 ^c^	0.09 ± 0.02 ^b^	0.08 ± 0.01 ^b^	0.08 ± 0.02 ^b^	0.45 ± 0.01 ^a^
Ethyl hexanoate	0.00 ± 0.00 ^c^	0.19 ± 0.01 ^a^	0.13 ± 0.03 ^b^	0.06 ± 0.01 ^c^	0.22 ± 0.03 ^a^
Isoamyl acetate	0.10 ± 0.00 ^d^	0.15 ± 0.00 ^c^	0.20 ± 0.01 ^b^	0.29 ± 0.01 ^a^	0.19 ± 0.01 ^b^
**Alcohols**					
n-Propanol	14.1 ± 0.3 ^d^	24.9 ± 0.8 ^ab^	26.5 ± 0.3 ^a^	22.6 ± 2.4 ^b^	17.8 ± 0.3 ^c^
Isobutanol	6.6 ± 0.2 ^d^	18.0 ± 0.9 ^ab^	19.2 ± 0.7 ^a^	16.4 ± 1.5 ^b^	9.5 ± 0.3 ^c^
Amylic alcohol	4.1 ± 0.1 ^d^	14.0 ± 0.03 ^a^	12.7 ± 0.2 ^b^	12.3 ± 0.1 ^b^	7.9 ± 0.2 ^c^
Isoamylic alcohol	30.2 ± 0.2 ^d^	56.1 ± 1.4 ^b^	61.9 ± 0.9 ^a^	56.3 ± 2.5 ^b^	37.23 ± 1.8 ^c^
β-Phenyl ethanol	4.53 ± 0.03 ^c^	6.44 ± 0.01 ^b^	6.16 ± 0.01 ^b^	4.25 ± 0.00 ^c^	7.29 ± 0.00 ^a^
**Carbonyl compounds**					
Acetaldehyde	82.4 ± 6.8 ^b^	38.5 ± 2.8 ^c^	102.6 ± 8.7 ^a^	93.0 ± 3.7 ^ab^	84.9 ± 7.03 ^b^
**Terpens**					
Linalool	0.09 ± 0.01 ^ab^	0.11 ± 0.02 ^a^	0.07 ± 0.01 ^bc^	0.05 ± 0.01 ^c^	0.11 ± 0.01 ^a^
α-terpineol	0.045 ± 0.002 ^bc^	0.034 ± 0.00 ^c^	0.134 ± 0.276 ^a^	0.042 ± 0.014 ^bc^	0.058 ± 0.001 ^b^
Citronellol	0.210 ± 0.01 ^e^	0.747 ± 0.007 ^b^	0.612 ± 0.007 ^c^	0.436 ± 0.00 ^d^	0.781 ± 0.014 ^a^

Data are the means ± standard deviation. Data with different superscript letters (^a,b,c,d^) within each row are significantly different (Duncan tests; *p* < 0.05).

**Table 4:** The main by-products and volatile compounds in the beers produced by *Z. florentina* in the pure and mixed fermentations (mg/L).

	*Z. florentina* DiSVA 263	US05 + DiSVA 263 1:1	US05 + DiSVA 263 1:10	US05 + DiSVA 263 1:20	*S. cerevisiae* US-05
**Esters**					
Ethyl butyrate	0.02 ± 0.014 ^c^	0.01 ± 0.00 ^c^	0.064 ± 0.014 ^b^	0.140 ± 0.014 ^a^	0.042 ± 0.014 ^b^
Ethyl acetate	1.6 ± 0.1 ^b^	8.33 ± 0.04 ^a^	7.7 ± 2.1 ^a^	6.7 ± 5.0 ^a^	2.6 ± 0.2 ^b^
Phenyl ethyl acetate	0.17 ± 0.017 ^d^	0.317 ± 0.01 ^b^	0.26 ± 0.0 ^c^	0,09 ± 0.02 ^b^	0.45 ± 0.01 ^a^
Ethyl hexanoate	0.00 ± 0.00 ^c^	0.09 ± 0.02 ^b^	0.08 ± 0.00 ^b^	0.02 ± 0.00 ^c^	0.22 ± 0.03 ^a^
Isoamyl acetate	0.30 ± 0.01 ^a^	0.34 ± 0.02 ^a^	0.23 ± 0.01 ^b^	0.33 ± 0.04 ^a^	0.190 ± 0.01 ^b^
**Alcohols**					
n-Propanol	14.0 ± 3.0 ^b^	19.44 ± 0.36 ^a^	18.9 ± 1.9 ^a^	19.9 ± 0.3 ^a^	17.8 ± 0.3 ^c^
Isobutanol	6.9 ± 3.1 ^b^	11.25 ± 1.34 ^a^	10.2 ± 0.3 ^ab^	11.8 ± 0.3 ^a^	9.5 ± 0.3 ^ab^
Amylic alcohol	3.49 ± 2.7 ^b^	7.85 ± 0.17 ^a^	7.9 ± 0.7 ^a^	7.8 ± 1.0 ^a^	7.9 ± 0.2 ^a^
Isoamylic alcohol	20.4 ± 13.4 ^c^	43.53 ± 3.21 ^a^	41.0 ± 1.6 ^a^	41.3 ± 0.3 ^a^	37.23 ± 1.8 ^bc^
β-Phenyl ethanol	1.74 ± 0.144 ^e^	3.94 ± 0.001 ^c^	4.17 ± 0.007 ^b^	2.78 ± 0.001 ^d^	7.29 ± 0.00 ^a^
**Carbonyl compounds**					
Acetaldehyde	14.2 ± 1.9 ^d^	51.63 ± 9.74 ^c^	56.9 ± 0.9 ^bc^	68.8 ± 0.014 ^b^	84.9 ± 7.03 ^a^
**Terpens**					
Linalol	0.051 ± 0.014 ^b^	0.124 ± 0.028 ^a^	0.129 ± 0.007 ^a^	0.041 ± 0.001 ^b^	0.114 ± 0.007 ^a^
α-terpineol	0.094 ± 0.028 ^a^	0.078 ± 0.006 ^a^	0.088 ± 0.001 ^a^	0.077 ± 0.015 ^a^	0.058 ± 0.001 ^a^
Citronellol	0.401 ± 0.020 ^c^	0.402 ± 0.073 ^c^	0.263 ± 0.022 ^b^	0.237 ± 0.026 ^d^	0.781 ± 0.014 ^a^

Data are the means ± standard deviation. Data with different superscript letters (^a,b,c,d^) within each row are significantly different (Duncan tests; *p* < 0.05).

**Table 5:** The main by-products and volatile compounds in the beers produced by *W. anomalus* in the pure and mixed fermentations (mg/L).

	*W. anomalus* DiSVA 2	US05 + DiSVA 2 1:1	US05 + DiSVA 2 1:10	US05 + DiSVA 2 1:20	*S. cerevisiae* US-05
**Esters**					
Ethyl butyrate	0.040 ± 0.042 ^c^	0.292 ± 0.021 ^a^	0.216 ± 0.008 ^b^	0.218 ± 0.007 ^b^	0.042 ± 0.014 ^c^
Ethyl acetate	1.7 ± 0.7 ^c^	15.7 ± 2.5 ^a^	9.6 ± 1.9 ^b^	7.5 ± 0.9 ^b^	2.6 ± 0.2 ^c^
Phenyl ethyl acetate	0.061 ± 0.035 ^d^	0.153 ± 0.001 ^cd^	0.360 ± 0.007 ^b^	0.230 ± 0.070 ^c^	0.452 ± 0.01 ^a^
Ethyl hexanoate	0.056 ± 0.014 ^c^	0.115 ± 0.007 ^b^	0.058 ± 0.014 ^c^	0.098 ± 0.001 ^b^	0.225 ± 0.029 ^a^
Isoamyl acetate	0.051 ± 0.049 ^c^	0.186 ± 0.021 ^b^	0.572 ± 0.735 ^a^	0.273 ± 0.004 ^b^	0.190 ± 0.014 ^b^
**Alcohols**					
n-Propanol	22.5 ± 0.9 ^a^	17.9 ± 0.7 ^c^	19.8 ± 0.5 ^b^	17.4 ± 0.5 ^c^	17.8 ± 0.3 ^c^
Isobutanol	10.8 ± 1.3 ^a^	7.6 ± 2.7 ^b^	8.7 ± 0.6 ^b^	8.4 ± 0.3 ^b^	9.5 ± 0.3 ^b^
Amylic alcohol	9.2 ± 0.5 ^a^	7.8 ± 1.4 ^a^	7.2 ± 0.1 ^a^	7.2 ± 1.8 ^a^	7.9 ± 0.2 ^c^
Isoamylic alcohol	51.6 ± 4.1 ^a^	35.0 ± 4.8 ^b^	38.4 ± 6.8 ^b^	32.0 ± 8.2 ^b^	37.23 ± 1.8 ^b^
β-Phenyl ethanol	0.00 ± 0.00 ^e^	4.70 ± 0.020 ^c^	6.14 ± 0.030 ^b^	3.50 ± 0.008 ^d^	7.29 ± 0.00 ^a^
**Carbonyl compounds**					
Acetaldehyde	49.3 ± 2.8 ^c^	57.0 ± 2.2 ^b^	51.00 ± 0.02 ^c^	26.8 ± 2.5 ^d^	84.9 ± 7.03 ^a^
**Terpens**					
Linalol	0.110 ± 0.010 ^a^	0.181 ± 0.039 ^a^	0.190 ± 0.029 ^a^	0.163 ± 0.006 ^a^	0.114 ± 0.007 ^a^
α-terpineol	0.022 ± 0.001 ^c^	0.130 ± 0.014 ^a^	0.062 ± 0.018 ^b^	0.088 ± 0.032 ^b^	0.058 ± 0.001 ^b^
Citronellol	0.00 ± 0.00 ^c^	0.00 ± 0.00 ^c^	0.155 ± 0.032 ^b^	0.165 ± 0.042 ^b^	0.781 ± 0.014 ^a^

Data are the means ± standard deviation. Data with different superscript letters (^a,b,c,d^) within each row are significantly different (Duncan tests; *p* < 0.05).
